# Genome-Wide Identification and Characterisation of the *4-Coumarate–CoA Ligase (4CL)* Gene Family in *Gastrodia elata* and Their Transcriptional Response to Fungal Infection

**DOI:** 10.3390/ijms26157610

**Published:** 2025-08-06

**Authors:** Shan Sha, Kailang Mu, Qiumei Luo, Shi Yao, Tianyu Tang, Wei Sun, Zhigang Ju, Yuxin Pang

**Affiliations:** 1 Pharmacy School, Guizhou University of Traditional Chinese Medicine, Guiyang 550025, China; sha18932003204@163.com (S.S.); mkl980818@163.com (K.M.); 19885611272@163.com (Q.L.); m19185010203@163.com (S.Y.); tang13984216529@163.com (T.T.); 2 College of Life Sciences, Guizhou Normal University, Guiyang 550025, China; sunwei889@163.com; 3 Gastrodia Elata Industrial Technology Research Institute of Guizhou Province, Guiyang 550004, China

**Keywords:** *Gastrodia elata* Blume, 4CL gene family, fungal infection, molecular characterisation, transcriptome analysis, molecular defence

## Abstract

*Gastrodia elata* Blume is an important medicinal orchid, yet its large-scale cultivation is increasingly threatened by fungal diseases. The 4-coumarate–CoA ligase (4CL) gene family directs a key step in phenylpropanoid metabolism and plant defence, but its composition and function in *G. elata* have not been investigated. We mined the *G. elata* genome for 4CL homologues, mapped their chromosomal locations, and analysed their gene structures, conserved motifs, phylogenetic relationships, promoter cis-elements and codon usage bias. Publicly available transcriptomes were used to examine tissue-specific expression and responses to fungal infection. Subcellular localisation of selected proteins was verified by transient expression in *Arabidopsis* protoplasts. Fourteen Ge4CL genes were identified and grouped into three clades. Two members, Ge4CL2 and Ge4CL5, were strongly upregulated in tubers challenged with fungal pathogens. Ge4CL2 localised to the nucleus, whereas Ge4CL5 localised to both the nucleus and the cytoplasm. Codon usage analysis suggested that *Escherichia coli* and *Oryza sativa* are suitable heterologous hosts for Ge4CL expression. This study provides the first genome-wide catalogue of 4CL genes in *G. elata* and suggests that Ge4CL2 and Ge4CL5 may participate in antifungal defence, although functional confirmation is still required. The dataset furnishes a foundation for functional characterisation and the molecular breeding of disease-resistant *G. elata* cultivars.

## 1. Introduction

*Gastrodia elata* Blume (Orchidaceae) is a prized traditional medicinal herb distributed chiefly in Guizhou, Yunnan, Jilin, Sichuan, and neighbouring provinces of China, and it is now listed as a nationally protected rare species [1,2]. Its tubers contain abundant bioactive constituents—phenols, flavonoids, polysaccharides, sterols, and others—that underpin diverse pharmacological activities [3,4,5,6]. Gastrodin, the principal phenolic glycoside, exhibits anti-inflammatory, anticancer, antiviral, and neuroprotective effects and is widely prescribed for neurological, cardiovascular, and hepatic disorders [7]. Commercial cultivation of *G. elata* has expanded steadily since artificial propagation was achieved in the late twentieth century [8]. However, large-scale production is increasingly compromised by fungal diseases that flourish under the warm, humid conditions required for Tianma cultivation, causing serious yield and quality losses [9,10]. Breeding disease-tolerant cultivars therefore represents a pressing need and requires functional dissection of genes that orchestrate antifungal defence in this species.

The 4-coumarate–CoA ligase (4CL; EC 6.2.1.12) gene family is a pivotal branch-point in phenylpropanoid metabolism and plays multifaceted roles in plant–pathogen interactions [11,12,13,14,15,16]. Infection by *Pseudomonas syringae* pv. maculicola markedly upregulates At4CL transcripts in *Arabidopsis thaliana* [17]. Overexpressing *Gh4CL3* in cotton enhances lignin and flavonoid accumulation and confers systemic resistance to *Verticillium dahlia* [18], whereas ectopic expression of *Os4CL3* or *Os4CL5* in rice restricts *Magnaporthe oryzae* penetration by reinforcing early lignin deposition [19]. Genome-wide surveys have catalogued 4CL repertoires in numerous crops, including pomegranate [20], apple [21], cassava [22], *Populus trichocarpa* [23], and *A*. *thaliana* [24]. In contrast, investigations of *G. elata* have focused mainly on chemical diversity, pharmacological action, and functional food development [4,25]; comprehensive analyses of defence-related genes, including *4CLs*, remain scarce.

In this study, systematic identification and analysis of the members of the 4CL gene family were conducted based on the whole-genome data of *G. elata*. The molecular characteristics of the Ge4CLs were systematically analysed through bioinformatics methods. Meanwhile, the influencing factors of codon usage bias and the optimal host expression system of Ge4CL genes were predicted based on codon usage characteristics. Subsequently, the transcriptome data were used to further reveal the tissue-specific expression patterns of the Ge4CLs, as well as their expression patterns in the tubers of healthy and pathogenic fungal infective states. Two of the Ge4CLs, Ge4CL2 and Ge4CL5, were verified through subcellular localisation experiments. This study systematically clarifies the evolutionary characteristics and functional diversity of the 4CL gene family in *G. elata* and lays a foundation for the functions of the 4CL gene family, which provides key candidate genes for cultivating *G. elata* with strong antibacterial properties.

## 2. Results

### 2.1. Identification and Chromosomal Location Analysis of G. elata Ge4CL Gene Family Members

**Figure 1:**
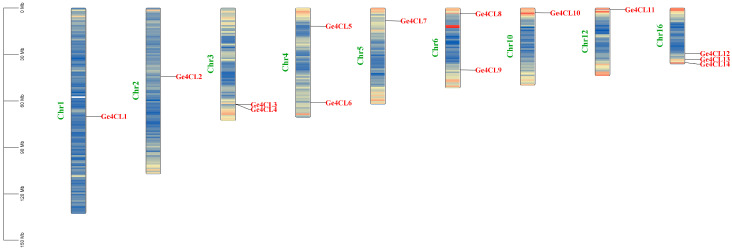
Physical locations of *Ge4CLs* in *G. elata*: Chromosome numbers are marked on the left side of the chromosomes in green font, and chromosome lengths are indicated in megabases (Mb). The red colours on the chromosomes represent the highest density, and blue indicates the lowest density.

Fourteen non-redundant 4CL genes (*Ge4CL1–Ge4CL14*) were recovered from the *G. elata* genome after BLASTP screening, Pfam domain confirmation, and AMP-binding domain verification with NCBI-CDD. These genes map to nine chromosomes (Figure 1). Chromosome 16 houses the largest cluster—*Ge4CL12*, *Ge4CL13*, and *Ge4CL14*—whereas chromosomes 2, 4, 5, 7, and 13 each contain a single locus. A tandem-duplication pair (*Ge4CL3*/*Ge4CL4*) was detected on chromosome 3; no additional tandem arrays were observed. The predicted protein lengths span 522–697 amino acids, corresponding to molecular masses of 55.7–77.4 kDa. The mean isoelectric point is 6.6, and the instability indices range from 27.81 to 49.47, classifying most members as stable (Table 1). The grand average of hydropathicity (GRAVY) values exceed 0 for all but *Ge4CL2*, *Ge4CL8*, *Ge4CL11*, and *Ge4CL12*, indicating that most isoforms are hydrophobic. PSORT analysis predicts primary localisation in the cytoplasm and plasma membrane, with subsidiary targeting to chloroplasts and the endoplasmic reticulum.

### 2.2. Collinearity Analysis

**Figure 2:**
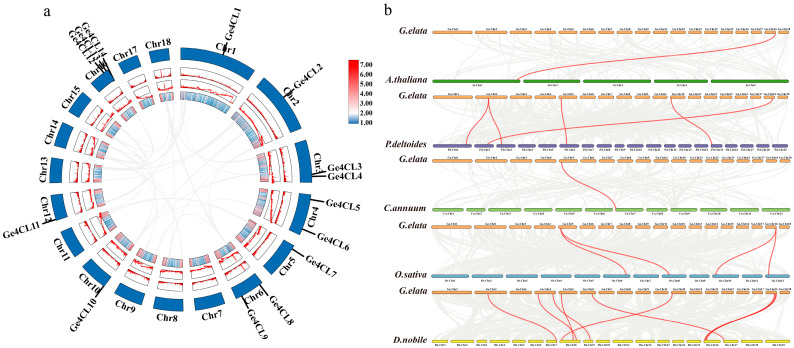
Collinearity analysis of *Ge4CLs* in *G. elata*: (**a**) Intra-species collinearity analysis of *Ge4CLs*. (**b**) Collinearity analysis of *4CLs* between *G. elata* and five other representative plants. The grey lines represent collinear blocks, and the red lines represent collinear *4CL* gene pairs.

To trace the origin and evolution of the *Ge4CL* family, we analysed both intra- and inter-species synteny. No segmental or tandem-duplication events were detected among the 14 loci within the *G. elata* genome (Figure 2a), indicating that the family size has remained stable during long-term evolution. Comparative mapping between *G. elata* and five reference genomes—*Arabidopsis thaliana*, *Populus trichocarpa*, *C*. *annuum*, *O*. *sativa*, and *D*. *officinale*—revealed the strongest collinear relationships with the fellow orchid *D. officinale* (Figure 2b). Orthology with *A. thaliana* and *C. annuum* was limited, reflecting lineage-specific divergence and genomic rearrangement.

### 2.3. Phylogenetic Tree Analysis

**Figure 3:**
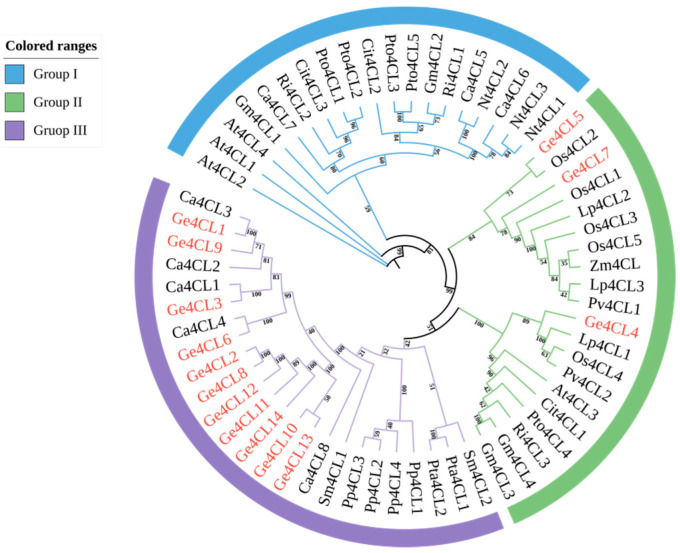
Maximum-likelihood (ML) phylogenetic tree of 4-coumarate–CoA ligase (4CL) proteins from *G. elata* (Ge) and 43 reference plant species. The tree was reconstructed in TBtools v1.138 using the JTT + G4 model and 5000 bootstrap replicates; bootstrap support values (%) are shown at the nodes. Subfamilies I–III are colour-shaded for clarity. Protein IDs of reference sequences are given in parentheses (e.g., At4CL1 = *A*. *thaliana* At1g51680; Os4CL1 = *O*. *sativa* AK069932.1; full list provided in Materials and Methods). Scale bar indicates the number of substitutions per site.

A maximum-likelihood tree constructed from 63 plant 4CL proteins divided all members into three clades (Figure 3). *Ge4CL4*, *Ge4CL5*, and *Ge4CL7* were grouped with *At4CL3* in clade II, which participates in flavonoid biosynthesis. Most *Ge4CL* genes were clustered in clade III together with homologues from *C. annuum*, *Selaginella moellendorffii*, *Physcomitrella patens*, and *Pinus* spp., suggesting a shared evolutionary trajectory. None of the fourteen *Ge4CL* proteins were grouped with the canonical lignin-associated Group I defined by Endler et al. Two members (*Ge4CL8* and *Ge4CL12*) were clustered within the flavonoid-associated Group II, whereas the remaining twelve proteins resided in the monocot-specific Group III previously reported for rice by Gui et al. This distribution indicates a lineage-specific expansion of Group III isoforms in *G. elata*.

### 2.4. Analysis of Conserved Motifs and Gene Structures

**Figure 4:**
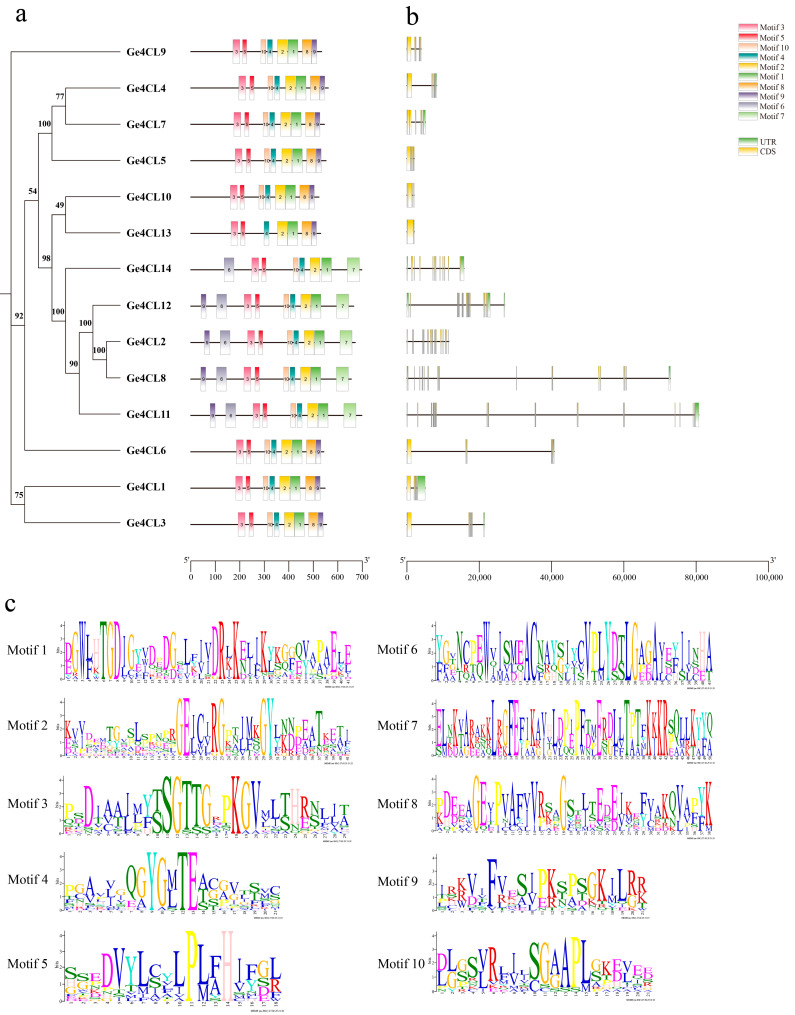
Conserved motifs and gene structure analysis: (**a**) Conserved motifs of *Ge4CLs*. (**b**) Gene structures of *Ge4CLs*. (**c**) Motif identifications of *Ge4CLs*.

Ten conserved motifs (Motif 1–Motif 10) were detected across all 14 Ge4CL proteins (Figure 4a). The study found that the *Ge4CLs* gene family has highly conserved functional domains. Each gene contains Motif 2 and Motif 3. Among them, Motif 3 contains an AMP-binding functional domain (SSGTTGLPKGV), and Motif 2 contains a conserved domain (GEICIRG) (Figure 4c). Gene structure analysis shows that all *Ge4CL* genes contain exons, but the number of exons varies to some extent (Figure 4b). Moreover, the intron lengths of *Ge4CL8* and *Ge4CL11* are longer than those of the other *Ge4CLs*. These results provide molecular evolutionary evidence for explaining the functional differentiation of *Ge4CLs*.

### 2.5. Analysis of Promoter Cis-Acting Elements

**Figure 5:**
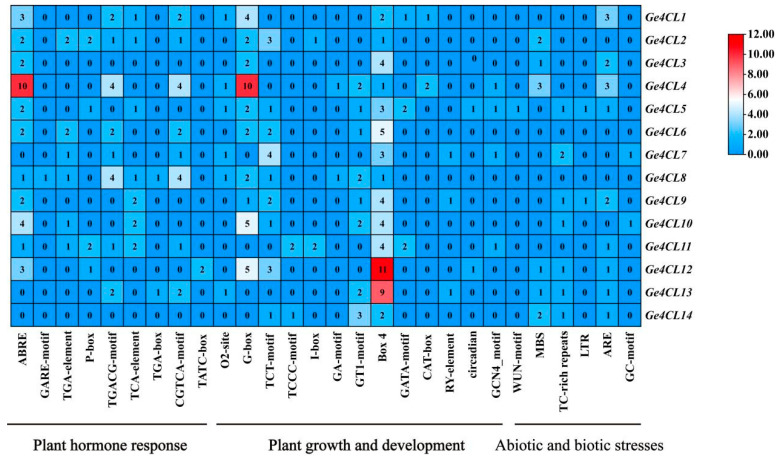
Distribution of different types of *cis*-acting elements in *Ge4CLs*: The numbers in the heatmap represent the quantities of different types of *cis*-acting elements in the promoter regions.

Promoter scanning uncovered 28 *cis*-elements that fall into three functional categories: hormonal response (e.g., ABA- and MeJA-responsive motifs), growth and development (light-responsive elements), and abiotic/biotic stress (drought- and defence-related motifs) (Figure 5). The heterogeneous distribution of these elements suggests that individual *Ge4CL* genes participate in distinct regulatory networks across development and stress adaptation. Notably, several defence-associated motifs were detected. The classic W-box (TTGACC/T), a binding site for WRKY transcription factors involved in fungal resistance, occurs in the promoters of *Ge4CL2* and *Ge4CL5*. We also identified the TC-rich repeat (ATTTTCTTCA), which is linked to stress-responsive gene activation. In the hormone-responsive category, MeJA-associated CGTCA/TGACG motifs and ethylene-responsive elements (EREs) are widely distributed, whereas multiple ABRE sites suggest ABA-regulated transcription. The co-presence of W-box and MeJA/ethylene motifs in *Ge4CL2* and *Ge4CL5* aligns with their strong induction upon fungal infection, implying that these genes may be jointly regulated by hormone-mediated defence pathways.

### 2.6. Analysis of Codon-Related Parameters of the Gene Family

The coding sequence (CDS) of the *Ge4CL* gene family was analysed with CodonW and EMBOSS-CUSP (Table 2). At the third codon position, the average nucleotide frequencies were 23% A, 29% T, 37% C, and 33% G. The GC contents at each codon position were not evenly distributed, which is consistent with the rules of monocotyledonous plants. The total GC content of *Ge4CLs* ranges from 41% to 66%, while the average ranges of GC1, GC2, and GC3 are 56.11%, 43%, and 58%, respectively. The codon adaptation index (CAI) ranges from 0.18 to 0.23; the codon bias index (CBI) ranges from −0.07 to 0.14; the frequency of optimal codons (FOP) ranges from 0.35 to 0.49; the effective number of codons (ENC) ranges from 41.15 to 56.44, and all genes have a value greater than 35, indicating a weak codon bias; the number of synonymous codons (L_sym) ranges from 506 to 677; and the aromaticity of protein (Aromo) ranges from 0.06 to 0.11.

### 2.7. Analysis of Influencing Factors on Codon Usage Bias of the Gene Family

**Figure 6:**
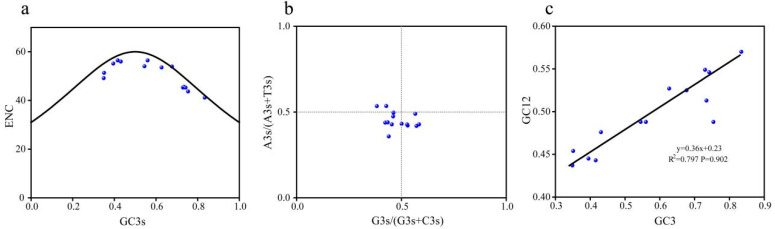
Analysis of codon usage bias of the *Ge4CLs*: (**a**) ENC plot analysis. GC3s: GC content at the third position of the codon; (**b**) PR2 plot analysis. A3s: content of base A at the third position of the codon; T3s: content of base T at the third position of the codon; G3s: content of base G at the third position of the codon; C3s: content of base C at the third position of the codon. In the figure, x = 0.5 and y = 0.5 are used as reference lines for analysing codon preference. (**c**) Neutral plot analysis. GC3: GC content at the third position of the codon; GC12: average GC content at the first and second positions of the codon.

ENC plot analysis (Figure 6a) placed two *Ge4CL* genes near the Wright expected curve, whereas the remaining members fell below it. The overall distribution followed the theoretical trend, indicating that mutation pressure largely shapes codon choice, with natural selection acting on a subset of genes. PR2 plot analysis (Figure 6b) revealed a slight preference for pyrimidines (T + C) over purines (A + G) at third-codon positions, supporting unequal mutation pressure. Neutral plot regression of GC12 against GC3s produced a slope of 0.36 (r = 0.89; Figure 6c), highlighting natural selection as the primary driver of codon usage, supplemented by mutation effects.

### 2.8. Selection of the Recipient System of the Ge4CLs

Because an efficient genetic transformation system for *G. elata* is not yet available, heterologous hosts were evaluated by comparing codon usage patterns. Codons exhibiting significant bias (ratio ≤ 0.5 or ≥2.0) between *Ge4CL* genes and the genomes of five model organisms were counted (Table 3). The results showed that the numbers of sites with significant differences in codon usage bias between the *Ge4CL* genes and the genomes of *Escherichia coli* and *Saccharomyces cerevisiae* were 10 and 20, respectively. This indicates that *Escherichia coli* is more suitable as a prokaryotic expression vector for the *Ge4CL* genes. When using *S. cerevisiae* as a eukaryotic expression system, the codons of the *4CLs* need to be optimised. In addition, the numbers of sites with significant differences in codon bias between *G. elata* and *A. thaliana*, *Nicotiana tabacum*, and *Oryza sativa* were 7, 16, and 1, respectively. The results showed that *O. sativa* was more suitable for the genetic transformation of the *Ge4CLs*.

### 2.9. Expression Patterns of the Ge4CLs in G. elata

**Figure 7:**
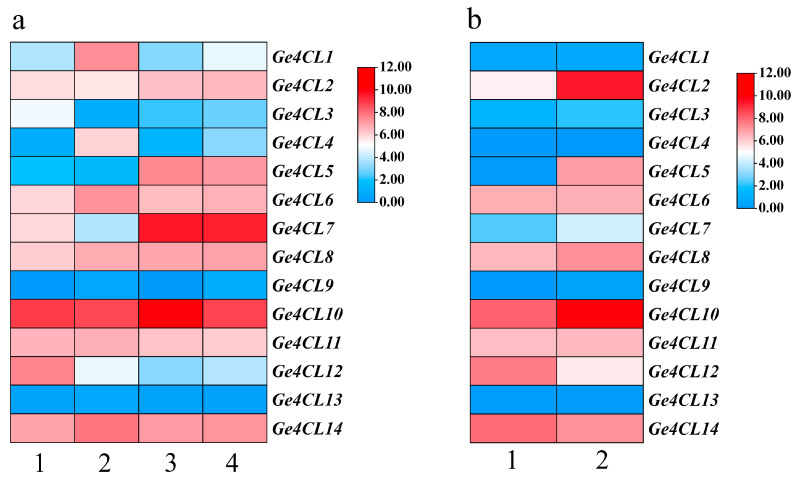
Expression profiles of *Ge4CL* genes based on public RNA-Seq data: (**a**) Tissue specificity across four developmental tissues: 1 = mature tuber (MT), 2 = juvenile tuber (JT), 3 = mother tuber of *G. elata* (MT-Ge), 4 = mother tuber of juvenile (MT-JT). (**b**) Comparison between fungal-diseased (FD) and healthy (HT) mature tubers of *G. elata f. glauca*. Heatmaps were generated from log2-transformed TPM values; colour scale ranges from red (high expression) to blue (low expression). Gene IDs are listed on the left.

RNA-Seq analysis (Figure 7a) revealed that most *Ge4CL* transcripts accumulate in mother tubers—both mature and juvenile—while showing moderate levels in daughter tubers. *Ge4CL9* and *Ge4CL13* were scarcely expressed in any tissue (TPM < 1). In contrast, *Ge4CL10* maintained high expression across all four tissues (TPM > 8), implying a broad role in phenylpropanoid biosynthesis. Under pathogenic fungal infection (Figure 7b), *Ge4CL2* and *Ge4CL5* were significantly upregulated, suggesting that these genes help orchestrate defence-related secondary metabolite production.

### 2.10. Subcellular Localisation Analysis

**Figure 8:**
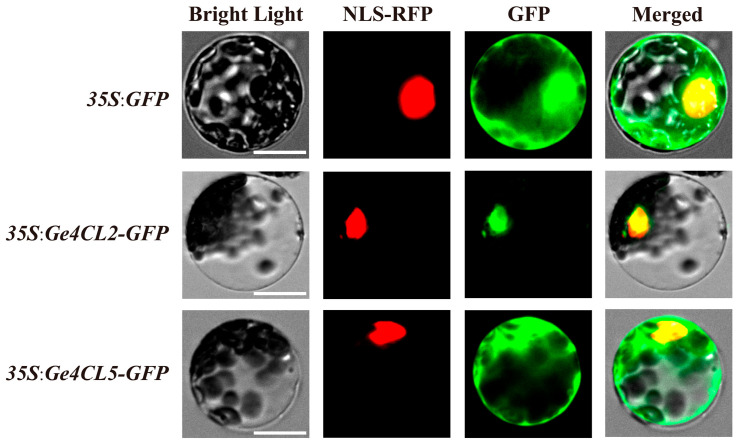
Subcellular localisation analysis of Ge4CL2 and Ge4CL5 proteins: Bright Light: The field of view under the bright-light channel, showing the cell morphology. NLS-RFP: The field of view under the red fluorescence channel; NLS-RFP was localised in the nucleus. GFP: The field of view under the green fluorescence channel, showing the localisation of the green fluorescent protein (GFP). Merged: The overlapping effect of the images under the three channels. The scale bar is 25 nm.

To confirm subcellular distribution, the CDS fragments of *Ge4CL2* and *Ge4CL5* (lacking stop codons) were fused to GFP in pUC19 and transiently co-expressed with an *NLS-RFP* marker in *Arabidopsis* protoplasts. Confocal microscopy 18 h post-transformation (Figure 8) showed *Ge4CL2-GFP* fluorescence confined to nuclei, whereas *Ge4CL5-GFP* signals were detected in both nuclei and cytoplasm, consistent with nuclear localisation for *Ge4CL2* and dual nuclear–cytoplasmic localisation for *Ge4*.

## 3. Discussion

### 3.1. Evolutionary Expansion and Diversification of the Ge4CL Gene Family in G. elata

The identification of 14 distinct *4CL* genes in *G. elata* represents a substantial expansion of the *4CL* gene family compared to model plants such as *Arabidopsis thaliana* (4 genes) and *Oryza sativa* (5 genes) [20,26]. However, this is fewer than in species like *Malus domestica* (69 genes) and *Gossypium hirsutum* (34 genes), suggesting that the family in *G. elata* is both relatively small and distinctively shaped by its evolutionary pressures [17,27]. The phylogenetic analysis of these genes indicated their clear grouping into three distinct subfamilies, highlighting functional divergence [28]. While subfamily I is typically involved in the biosynthesis of lignin, a key secondary metabolite in many plants, *G. elata*’s *4CL*s are not found in this subfamily. This absence supports the hypothesis that *G. elata*’s *4CL*s have evolved to play a different role in secondary metabolism, perhaps involved in the biosynthesis of compounds like flavonoids, which are critical for its medicinal properties. Functional implications of the Group III enrichment: Dicots typically harbour two 4CL subclasses, with Group I enzymes channelling precursors into lignin and Group II enzymes fuelling flavonoid formation. A third subclass, Group III, appears to be restricted to monocots and is thought to support stress-responsive phenylpropanoid branches rather than structural lignification. The fact that twelve out of fourteen *Ge4CL* genes fall into Group III, while none remain in Group I, suggests that lignin-directed CoA ligase activity may be complemented by other acyl-activating enzymes in this achlorophyllous orchid; conversely, the expansion of Group III members could confer metabolic flexibility, allowing rapid redirection of phenylpropanoid flux toward antifungal defence or symbiotic interactions during subterranean growth. Biochemical assays and reverse-genetics studies will be required to verify whether individual Ge4CL isoforms specialise in these adaptive pathways.

Notably, the occurrence of tandem duplications on chromosome 3 could suggest that gene expansion in this species was driven by such duplications, a process commonly observed in other plant species (and particularly in plants that undergo selective pressure for pathogen resistance) [29]. These results offer important insights into the evolutionary forces that have shaped the *4CL* family in *G. elata*, and potentially in other orchids or medicinal plants with similar evolutionary histories. Further comparison with the *4CL* family in other medicinal plants may reveal species-specific adaptations in the synthesis of bioactive secondary metabolites. It is well documented that the biosynthesis of flavonoids, phenolic acids, and other secondary metabolites contributes significantly to the medicinal and therapeutic properties of plants [30,31]. Given that *G. elata* is used in traditional medicine for its antioxidant and anti-inflammatory properties, the absence of *4CL* genes related to lignin biosynthesis but the presence of genes potentially involved in flavonoid synthesis could directly link the gene family’s evolution to the plant’s medicinal qualities. Moreover, the unique evolutionary trajectory of these genes in *G. elata* opens new avenues for the study of gene family diversification in other plants that may also possess therapeutic value, but with distinct metabolic pathways or molecular adaptations tailored to their ecological niches.

### 3.2. Role of Ge4CL Genes in Fungal Infection Response and Plant Defence Mechanisms

The expression analysis of *Ge4CL* genes in response to fungal infection in *G. elata* provided compelling evidence that certain members of the *4CL* gene family, particularly *Ge4CL2* and *Ge4CL5*, play an important role in the plant’s defence mechanism. These genes exhibited significant upregulation in infected tissues, suggesting their involvement in the plant’s antifungal response [32,33,34]. This aligns with findings in other species, where *4CL* genes have been linked to the biosynthesis of secondary metabolites like phenylpropanoids, which are well known to participate in plants’ defence against pathogens [20,35,36,37]. In particular, phenolic compounds such as flavonoids and lignin derivatives can act as physical barriers or antimicrobial agents to thwart pathogen invasion [38,39]. The upregulation of *Ge4CL2* and *Ge4CL5* in response to fungal stress might therefore indicate that these genes contribute to the synthesis of such defence compounds, reinforcing the plant’s ability to resist fungal infections.

In addition to expression data, the subcellular localisation of *Ge4CL2* and *Ge4CL5* further supports their roles in plant defence. *Ge4CL2*’s predominant localisation to the nucleus suggests that it might be involved in the regulation of gene expression related to stress responses, potentially through interactions with transcription factors that modulate defence pathways. On the other hand, the dual localisation of *Ge4CL5* in both the nucleus and cytoplasm hints at a broader functional scope, possibly including the regulation of metabolic pathways in addition to its involvement in defence. This dual localisation is significant, as it may indicate *Ge4CL5*’s participation in both transcriptional regulation and post-translational modifications, which are crucial for fine-tuning the plant’s response to external stressors such as fungal pathogens. These findings not only emphasise the central role of *Ge4CL* genes in stress responses but also open the door to further investigations into the precise biochemical and molecular pathways through which these genes contribute to *G. elata*’s antifungal properties.

### 3.3. Codon Usage Bias and Its Implications for Gene Expression in G. elata

The codon usage bias analysis conducted on the *Ge4CL* gene family provided insightful data on the evolutionary pressures shaping these genes [40]. The results indicated that the coding sequences of these genes exhibit a distinct bias, influenced by both mutational pressure and natural selection. This suggests that the *Ge4CL* genes in *G. elata* have undergone selective evolutionary pressures, likely driven by the plant’s adaptation to specific environmental conditions, including pathogen stress [41]. Interestingly, the codon usage patterns observed in *G. elata*’s *4CL* genes were comparable to those in other well-studied plants such as *Oryza sativa* and *Arabidopsis thaliana*, which further supports the hypothesis that codon usage bias is a general feature of plant gene families, influenced by both evolutionary history and environmental adaptation. Moreover, this codon usage bias could have implications for the efficient expression of these genes, suggesting that *G. elata*’s *4CL* genes are optimised for the particular ecological pressures that the plant faces.

Biological relevance of codon usage metrics: The 14 *Ge4CL* genes display medium codon adaptation indices (CAI = 0.18–0.23), suggesting a balanced usage that avoids excessive competition for tRNA pools. Notably, *Ge4CL2* and *Ge4CL5*—the two transcripts most strongly induced upon *Penicillium* infection—lie at the upper end of this range, indicating that their codon composition closely matches that of high-expression housekeeping genes and, thus, favours rapid translation under stress. The lower effective number of codons for Ge4CL5 (ENC = 43.68) further points to a restricted set of preferred codons, likely paired with abundant tRNAs, which can accelerate elongation and boost the flux toward defence-related phenylpropanoid metabolites. In contrast, *Ge4CL1*, *Ge4CL10*, and *Ge4CL13* exhibit GC contents > 0.60. Elevated GC may stabilise secondary mRNA structure while slightly lowering initiation efficiency, a feature congruent with their putative housekeeping or metabolic “buffer” roles under non-stress conditions. Together, these metrics suggest that codon usage in *G. elata* fine-tunes translational output, allowing rapid upregulation of key defence genes without globally perturbing protein synthesis homeostasis.

### 3.4. Evolutionary Implications of the Reduced 4CL Repertoire in G. elata

The present study identified only seven canonical *Ge4CL* genes, a copy number that lies at the lower end of the spectrum reported for angiosperms. Autotrophic species such as *Arabidopsis thaliana*, *Zea mays*, and *Oryza sativa* typically harbour 4–5 members, whereas lineage-specific duplications in woody or fruit crops can inflate the family to dozens of loci (e.g., 30 4CL/ACS-related genes in pear) [42]. Within Orchidaceae, the fully photosynthetic epiphyte *Vanilla planifolia* retains five 4CL genes [43], while draft genomes of *Dendrobium* spp. annotate ≈ 9–11 copies.

The intermediate yet streamlined complement in the achlorophyllous, fully mycoheterotrophic *G. elata* is consistent with relaxed selection on phenylpropanoid-derived lignin following the loss of leaves and the concomitant reduction in structural carbon demand. Comparative genomics of other mycoheterotrophic orchids and parasitic plants reveals parallel contractions in plastid and nuclear gene sets once photosynthesis is abandoned [44]. We therefore hypothesise that the modest downsizing of the 4CL family in *G. elata* reflects a balance between (i) conserving minimal enzymatic capacity for specialised metabolites such as gastrodin and (ii) genome-wide pressures to economise redundant functions. Testing this hypothesis will require broader taxon sampling (e.g., *Epipogium*, *Cuscuta*) and functional interrogation of lineage-specific 4CL paralogues via CRISPR knockout, enzyme activity assays, and targeted metabolomics, thereby clarifying whether convergent gene family contraction is a common hallmark of heterotrophic evolution.

### 3.5. Future Directions: Functional Validation and Applications in Plant Breeding

While this study provides a comprehensive genomic and bioinformatic characterisation of the *4CL* gene family in *Gastrodia elata*, the functional validation of these genes remains a critical next step. Future research should focus on experimental approaches to confirm the roles of *Ge4CL2* and *Ge4CL5* in the biosynthesis of secondary metabolites involved in defence responses. Techniques such as quantitative real-time PCR (qRT-PCR) and RNA interference (RNAi) could be used to validate the expression patterns of these genes under different stress conditions, and to explore their precise involvement in fungal resistance. Additionally, gene knockout or overexpression systems, coupled with metabolic profiling, could help identify the specific metabolites produced by these genes and their role in plant defence.

Beyond functional validation, our transcriptome-based findings already hint at tangible avenues for the molecular breeding of *G. elata* varieties with improved resistance to fungal diseases. Pinpointing *Ge4CL2* and *Ge4CL5* as putative defence-related genes suggests that they could be harnessed—either through marker-assisted selection or targeted engineering—to boost phenylpropanoid-derived metabolites and thereby enhance both plant vigour and medicinal quality. Such insights may, in turn, stimulate parallel efforts in other medicinal species, ultimately expanding the repertoire of crops that combine strong disease resistance with high bioactive compound yields. We recognise, however, that these proposals rest on correlative transcriptome evidence. Owing to the current unavailability of mature tuber material and pending biosafety approval for *Penicillium oxalicum* inoculation, qRT-PCR and functional complementation assays could not be completed within the present revision cycle. We have therefore scheduled controlled infection experiments for the coming harvest season and secured funding to perform qRT-PCR validation and enzyme assays. Until those data are obtained, all functional inferences should be regarded as hypotheses that require experimental confirmation.

## 4. Materials and Methods

### 4.1. Identification and Chromosomal Localisation of Ge4CL Genes

Whole-genome sequence data for *Gastrodia elata* (assembly accession GWHBDNU00000000, National Genomics Data Center, CN) were downloaded and screened for 4-coumarate–CoA ligase (4CL) candidates. The reference set of *Arabidopsis thaliana* 4CL proteins (TAIR; https://www.arabidopsis.org/, accessed on 8 December 2024) was first queried against the *G. elata* proteome with BLASTP (E-value < 1 × 10^−5^). In parallel, the hidden Markov model for the AMP-binding/4CL domain (Pfam entry PF00501) was used in HMMER 3.3 searches. Sequences lacking the conserved AMP-binding motif were discarded after confirmation with the NCBI Conserved Domain Database. Genomic coordinates for the retained genes were extracted from the *G. elata* GFF3 annotation and visualised with TBtools. Basic physicochemical properties—including amino acid length, predicted molecular weight, and isoelectric point—were calculated with the same software, while subcellular localisation was predicted using PSORT (https://wolfpsort.hgc.jp/, accessed on 10 December 2024).

To relate gene position to expression behaviour, we re-examined two publicly available RNA-Seq datasets originating from the same geographical provenance (Changbai Mountains, Jilin, China). Healthy mature tubers (BioSample SAMN14380862) and naturally *Penicillium oxalicum*-infected mature tubers (BioSample SAMN14380861) of *G. elata* Bl. f. glauca were collected on the same day, surface-sterilised, and dissected at identical anatomical sites. Each condition comprised three biological replicates (≈100 mg tissue per replicate), immediately flash-frozen in liquid nitrogen. Illumina NovaSeq sequencing yielded 7.89 × 10^10^ and 6.45 × 10^10^ clean bases for the healthy and diseased groups, respectively. Raw reads were quality-filtered with FastQC and Trimmomatic, aligned to the reference genome with HISAT2, and normalised counts were generated in DESeq2. Genes with |log_2_FC| ≥ 1 and FDR < 0.05 were considered to be differentially expressed. Concordance among biological replicates was high (Pearson r^2^ = 0.96–0.98), underscoring the reliability of the expression data used in subsequent analyses.

BLASTP searches were performed with NCBI BLAST+ v2.10.1, using the four annotated 4CL proteins of *Arabidopsis thaliana* (AT1G51680, AT3G21240, AT1G65060, and AT3G21230) as queries. The search parameters were set to E-value < 1 × 10^−5^, word size 3, BLOSUM62 matrix, gap open 11, gap extend 1, and low-complexity filtering disabled (-seg no); the maximum target sequences option was raised to 5000 to avoid premature truncation. In parallel, HMMER v3.3.2 was run against the *G. elata* proteome using the Pfam profile PF00501 (AMP-binding/4CL domain). The union of BLASTP and HMMER hits was purged of redundancy and screened in the NCBI Conserved Domain Database; proteins lacking the diagnostic AMP-binding motif (cl00909) were discarded.

### 4.2. Collinearity Analysis of the Ge4CL Gene Family

Chromosome length, gene coordinates, and gene density information were parsed from the *G. elata* genome and its GFF3 file. Intragenomic synteny was illustrated with the Advanced Circos function in TBtools. To examine inter-species collinearity, genome assemblies and annotations for *A. thaliana*, *P. trichocarpa*, *C. annuum*, *O. sativa*, and *D. officinale* were downloaded from NCBI and analysed in TBtools.

### 4.3. Phylogenetic Analysis of the Ge4CLs

Published 4CL amino acid sequences from diverse plant species were aligned, and a maximum-likelihood phylogenetic tree was built with the “One-Step Build a ML Tree” module in TBtools. Ultrafast bootstrap resampling (5000 replicates) was applied. The tree was refined in iTOL.

### 4.4. Conserved Motifs and Gene Structures of Ge4CLs

Conserved motifs were identified with MEME (http://meme-suite.org/, accessed on 12 December 2024), with the maximum number of motifs set to 10. Gene structures and motif distributions were visualised in TBtools alongside the phylogenetic tree.

### 4.5. Analysis of Cis-Acting Elements in the Promoters of Ge4CLs

Promoter regions (2 kb upstream of the ATG start codon) were extracted in TBtools. Cis-regulatory elements were predicted with PlantCARE, and their abundance was displayed as a heatmap generated in TBtools.

### 4.6. Analysis of Codon Usage Bias in the Ge4CLs

Codon composition and related parameters were calculated with CodonW and the CUSP program. The effective number of codons (ENC), the GC content at each codon position, and the frequency of A, T, C, and G at the third position of synonymous codons were determined. ENC plots, PR2 plots, and neutral plots were produced in Origin 2024.

### 4.7. Data Sources for Codon Usage Bias

Codon usage tables for Ge4CL genes were generated with the EMBOSS Explorer interface (http://www.bioinformatics.nl/emboss-explorer/, accessed on 13 December 2024). Genome-wide codon usage data for five model organisms—*E*. *coli*, *N*. *tabacum*, *A*. *thaliana*, *S*. *cerevisiae*, and *O*. *sativa*—were downloaded from the Kazusa Codon Usage Database (http://www.kazusa.or.jp/codon/, accessed on 13 December 2024). The codon usage metrics for each Ge4CL were compared with the genomic averages of the five reference organisms.

### 4.8. Analysis of Gene Family Expression Patterns

RNA-Seq datasets covering four *G. elata* tissues—mature tuber, juvenile tuber, mother tuber, and mother-of-juvenile tuber (SRA project SRP279888)—together with mature tubers showing fungal disease symptoms and healthy controls (SRP268570), were obtained from the GelFAP v2.0 portal. Transcript abundance was expressed as log_2_(TPM + 1). Expression heatmaps were drawn in TBtools.

Clean reads from the healthy and fungal-infected tuber libraries were processed in DESeq2 to obtain normalised gene-level counts. A gene was deemed to be significantly differentially expressed when it met both of the following criteria: (i) an absolute log_2_(fold change) ≥ 1, corresponding to a ≥2-fold up- or downregulation, and (ii) a Benjamini–Hochberg adjusted *p* value (FDR) < 0.05. Wald tests (pairwise contrasts) were used for two-group comparisons, while likelihood ratio tests provided ANOVA-like assessment in multi-group situations. All *p* values are two-tailed unless otherwise stated. The same thresholds were applied wherever ”significant expression change” is discussed in the Results section.

### 4.9. Cloning and Subcellular Localisation of Ge4CL2 and Ge4CL5

Coding sequences of *Ge4CL2* and *Ge4CL5* were PCR-amplified from *G. elata* cDNA with gene-specific primers lacking stop codons. Amplicons were purified, ligated into pUC19-GFP via seamless cloning, and transformed into *Escherichia coli* DH5α by heat shock. Positive clones were verified by Sanger sequencing (Kumei Biotechnology, Changchun, China), and endotoxin-free plasmids were prepared with the EndoFree Plasmid Midi Kit (CWBIO, Beijing, China). *pUC19-Ge4CL-GFP* and *NLS-RFP* constructs were co-transfected into *Arabidopsis thaliana* mesophyll protoplasts. After 18–22 h of dark incubation at 22 °C, GFP and RFP signals were visualised with a confocal laser scanning microscope to determine subcellular localisation.

## 5. Conclusions

In this study, we conducted a comprehensive genome-wide identification and characterisation of the *4CL* gene family in *G. elata*, revealing 14 distinct members with unique evolutionary and functional characteristics. Our findings indicate a possible role for Ge4CL genes, particularly *Ge4CL2* and *Ge4CL5*, in the plant’s response to fungal infection, suggesting their involvement in the biosynthesis of defence-related secondary metabolites. This work not only deepens our understanding of the molecular mechanisms underlying *G. elata’s* antifungal resistance but also lays the groundwork for future functional validation studies and potential applications in breeding *G. elata* varieties with enhanced disease resistance. The insights gained here contribute significantly to both the basic understanding of plant gene family evolution and the practical application of medicinal plant improvement.

## Figures and Tables

**Table 1:** Physicochemical properties of the Ge4CLs.

Gene Name	Gene ID	Number of aa	Molecular Weight/kD	pI Value	Instability Index	GRAVY	Subcellular Localisation
*Ge4CL1*	GelC01G00992.1	547	58.15	8.82	49.47	0.163	Plasma membrane
*Ge4CL2*	GelC02G00823.1	670	74.27	7.15	33.04	−0.064	Cytoplasm
*Ge4CL3*	GelC03G01279.2	553	60.16	8.51	41.85	0.095	Plasma membrane
*Ge4CL4*	GelC03G01284.1	560	60.16	6.29	35.89	0.179	Plasma membrane
*Ge4CL5*	GelC04G00359.1	551	60.10	5.42	43.37	0.06	Chloroplast
*Ge4CL6*	GelC04G01345.1	542	58.78	6.19	40.94	0.1	Plasma membrane
*Ge4CL7*	GelC05G00275.1	544	59.27	5.59	38.71	0.077	Plasma membrane
*Ge4CL8*	GelC06G00150.1	654	73.00	6.23	27.81	−0.081	Cytoplasm
*Ge4CL9*	GelC06G00789.1	533	57.28	6.47	44.49	0.166	Plasma membrane
*Ge4CL10*	GelC10G00141.1	522	55.70	5.88	42.5	0.022	Endoplasmic reticulum
*Ge4CL11*	GelC12G00053.2	697	77.41	6.53	34.69	−0.113	Cytoplasm
*Ge4CL12*	GelC16G00539.1	664	75.08	6.49	35.78	−0.191	Cytoplasm
*Ge4CL13*	GelC16G00628.1	529	57.48	6.34	36.82	0.043	Chloroplast
*Ge4CL14*	GelC16G00735.1	697	76.24	6.47	36.1	0.005	Plasma membrane

**Table 2:** Base composition of codons in the *Ge4CL* gene family.

Gene Name	T3s	C3s	A3s	G3s	GC3s	GC	GC1	GC2	GC3	CAI	CBI	Fop	ENC	L_sym	Aromo
*Ge4CL1*	0.15	0.53	0.17	0.33	0.73	0.61	0.61	0.49	0.73	0.21	0.14	0.49	45.27	531	0.07
*Ge4CL2*	0.43	0.24	0.32	0.27	0.42	0.43	0.51	0.37	0.42	0.20	−0.07	0.38	56.44	646	0.10
*Ge4CL3*	0.32	0.35	0.24	0.29	0.55	0.51	0.54	0.44	0.55	0.21	0.03	0.43	54.02	533	0.09
*Ge4CL4*	0.22	0.41	0.17	0.41	0.68	0.58	0.61	0.44	0.68	0.22	0.07	0.45	53.90	546	0.07
*Ge4CL5*	0.17	0.53	0.13	0.39	0.75	0.58	0.56	0.41	0.75	0.22	0.10	0.47	43.68	530	0.06
*Ge4CL6*	0.30	0.38	0.23	0.29	0.56	0.51	0.54	0.43	0.56	0.20	0.02	0.42	56.44	531	0.08
*Ge4CL7*	0.21	0.50	0.12	0.39	0.73	0.59	0.61	0.41	0.73	0.20	0.06	0.43	45.60	524	0.07
*Ge4CL8*	0.45	0.21	0.33	0.28	0.40	0.43	0.52	0.37	0.40	0.20	−0.06	0.38	55.16	634	0.11
*Ge4CL9*	0.22	0.40	0.22	0.35	0.63	0.56	0.58	0.47	0.63	0.21	0.13	0.48	53.56	518	0.06
*Ge4CL10*	0.16	0.48	0.15	0.41	0.74	0.61	0.61	0.48	0.74	0.23	0.13	0.49	45.28	513	0.07
*Ge4CL11*	0.47	0.18	0.35	0.25	0.35	0.42	0.48	0.42	0.35	0.20	−0.13	0.35	51.35	677	0.10
*Ge4CL12*	0.43	0.19	0.42	0.24	0.35	0.41	0.50	0.38	0.35	0.18	−0.12	0.35	49.18	639	0.11
*Ge4CL13*	0.09	0.56	0.11	0.42	0.83	0.66	0.65	0.49	0.83	0.20	0.10	0.46	41.15	506	0.07
*Ge4CL14*	0.41	0.25	0.30	0.28	0.43	0.46	0.53	0.42	0.43	0.21	−0.02	0.41	55.96	675	0.09

**Table 3:** Comparison of codon biases between the *Ge4CLs* and representative species.

Codon	AA	Ge/Ec	Ge/Nt	Ge/At	Ge/Sc	Ge/Os	Codon	AA	Ge/Ec	Ge/Nt	Ge/At	Ge/Sc	Ge/Os
UUU	Phe	0.86	0.84	0.97	0.81	1.61	UAU	Tyr	0.6	0.73	0.89	0.69	1.3
UUC		1.51	1.17	1.01	1.14	0.94	UAC		1.58	1.37	1.35	1.25	1.23
UUA	Leu	0.47	0.6	0.64	0.31	1.33	UAA	TER	0.1	0.18	0.22	0.18	0.29
UUG		1.21	0.7	0.75	0.57	1.06	UAG		2	1.2	1.2	1.2	0.75
CUU		1.21	0.73	0.73	1.42	1.15	CAU	His	0.85	0.78	0.76	0.77	0.93
CUC		3.07	2.37	1.81	5.4	1.13	CAC		1.34	1.13	1.13	1.26	0.71
CUA		1.34	0.8	0.76	0.56	0.97	CAA	Gln	0.68	0.47	0.99	0.36	0.73
CUG		0.57	2.09	2.17	2.03	1.01	CAG		0.56	1	0.99	1.24	0.72
AUU	Ile	0.85	0.91	1.18	0.84	1.78	AAU	Asn	0.58	0.6	0.76	0.47	1.12
AUC		1.3	1.82	1.37	1.47	1.3	AAC		0.67	0.77	0.66	0.55	1.74
AUA		1	0.95	1.06	0.75	1.51	AAA	Lys	0.56	0.63	0.67	0.49	1.29
AUG	Met	0.92	0.87	0.89	1.04	0.91	AAG		1.99	0.91	0.93	0.99	0.94
GUU	Val	1.06	0.86	0.85	1.04	1	GAU	Asp	0.82	0.75	0.75	0.73	1.09
GUC		1.79	2.1	1.83	1.98	1.16	GAC		1.14	1.21	1.19	1.01	0.73
GUA		0.71	0.82	0.94	0.79	1.37	GAA	Glu	0.77	0.75	0.79	0.59	1.25
GUG		1.16	1.38	1.33	2.14	0.95	GAG		1.67	0.8	1.01	1.69	0.84
UCU	Ser	1.20	0.79	0.62	0.67	1.24	UGU	Cys	1	0.6	1.56	0.73	0.95
UCC		2.11	2.01	1.83	1.44	1.26	UGC		2.29	1.75	1.75	2.63	1.02
UCA		0.89	0.66	0.64	0.63	0.94	UGA	TER	0.73	0.8	0.67	1.42	0.67
UCG		1.5	1.32	1.32	1.43	1	UGG	Trp	0.72	0.8	0.78	0.93	0.7
CCU	Pro	1.85	0.94	0.94	1.3	1.29	CGU	Arg	0.32	0.68	0.57	0.8	0.71
CCC		2.43	2.29	2.85	2.22	1.25	CGC		0.74	2.64	2.71	3.96	0.64
CCA		1.16	0.54	0.66	0.58	0.75	CGA		0.96	0.87	0.73	1.53	0.72
CCG		1.03	3	1.74	2.83	0.83	CGG		1.05	2.24	1.69	4.88	0.62
ACU	Thr	0.89	0.57	0.66	0.57	1.09	AGU	Ser	0.39	0.39	0.37	0.37	0.59
ACC		0.77	1.5	1.41	1.14	0.97	AGC		0.92	1.32	1.17	1.35	0.83
ACA		0.8	0.7	0.77	0.68	1.04	AGA	Arg	1.39	0.62	0.52	0.46	0.94
ACG		1.99	2.29	1.34	1.29	0.9	AGG		0.93	0.96	1.06	1.27	0.73
GCU	Ala	1.08	0.65	0.72	0.96	1.04	GGU	Gly	0.5	0.53	0.53	0.49	0.8
GCC		1.32	2.29	2.78	0.27	0.93	GGC		1.23	2.26	2.75	2.58	0.86
GCA		0.78	1.78	1.03	1.11	1.04	GGA		1.63	0.96	0.92	2.04	1.4
GCG		0.85	3.09	1.99	2.89	0.67	GGG		1.56	2.78	1.88	3.2	1.12

## Data Availability

The genome data of *Gastrodia elata* used in this study are publicly available from the National Genomics Data Center (NGDC) under accession number GWHBDNU00000000. Most of the transcriptome and gene expression data were obtained from the public database GelFAP v2.0 (http://www.gzybioinformatics.cn/Gelv2, accessed on 22 December 2024).

## Abbreviations

The following abbreviations are used in this manuscript:

4CL	4-Coumarate–CoA Ligase
Ge4CL	*Gastrodia elata* 4CL Gene
*G. elata*	*Gastrodia elata*
TPM	Transcripts Per Million
NGDC	National Genomics Data Center
CDD	Conserved Domain Database
MEME	Multiple EM for Motif Elicitation
ENC	Effective Number of Codons
CAI	Codon Adaptation Index
CBI	Codon Bias Index
FOP	Frequency of Optimal Codons
GC	Guanine–Cytosine Content
GFF	General Feature Format
PSORT	Protein Subcellular Localisation Prediction Tool
TBtools	Toolbox for Biologists
GFP	Green Fluorescent Protein
RFP	Red Fluorescent Protein
RNAi	RNA Interference
